# Bioprocessing and characterization of thermostable phytase from *Aspergillus terreus,* an endophyte of *Catharanthus roseus*, with a potential activity to hydrolyze phytic acid in wheat bran

**DOI:** 10.1186/s12896-025-00988-0

**Published:** 2025-07-08

**Authors:** Marwa A. Yassin, Eman H. Mohsen, Nelly M. George, Mohamed A. Marawan, Ashraf S. A. El-Sayed, Marwa M. El-Demerdash

**Affiliations:** 1 Enzymology and Fungal Biotechnology Lab, Botany and Microbiology Department, Faculty of Science, Zagazig University, Zagazig, 44519 Egypt; 2 Zoology Department, Faculty of Science, Mansoura University, Mansoura, Egypt

**Keywords:** *Aspergillus terreus*, Phytase, Phytic acid, Solid state fermentation, Wheat bran

## Abstract

Phytic acid is one of the common anti-nutritional factors in animal feeds, due to its chelating activity of metal ions and amino acids, so, phytase has been used for increasing the nutritional value of the animal feeds by releasing phosphorous. The stability and catalytic efficiency of this enzyme are the major challenges, so, the objective of this study was to purify and characterize phytase with relatively unique biochemical properties. Among the recovered fungal endophytes of *Catharanthus roseus, Aspergillus terreus* EFBL-AS PV412881.1 was recognized as the most potent phytase producing isolate. Upon nutritional optimization with the face-centered central composite design (FCCD), the productivity of phytase by *A. terreus* grown on wheat bran amended with 0.2% NaNO_3_ and 0.4 % yeast extract, under solid state fermentation, was increased into 36.3 μmol/mg/min. Phytase of *A. terreus* was purified to its molecular homogeneity by gel-filtration and ion-exchange chromatography, with 3.48 purification folds (125 μmol/mg/min). The purified enzyme had a molecular subunit 85 kDa by denaturing-PAGE, with highest activity at reaction temperature 37–40 °C, and reaction pH 7.0. The T_1/2_ of *A. terreus* phytase was 124.5, 5.2 and 3.8 h, at 4, 40, and 50 °C, respectively. The thermal denaturation rate (Kr) was 0.095 ×10^−3^, 0.27 × 10^−3^ and 0.292 ×10^−3^/min at 20, 40, and 50 °C, respectively. The enzyme was slightly inhibited by Ca^2+^ ions, unlike the resistance to various cations. The concentration of phytic acid of wheat bran was reduced by about 6.5 folds upon phytase treatment, ensuring the feasibility of this enzyme in the animal feed application. From the molecular docking analysis, phytase from *A. terreus* had a higher affinity to hydrolyze phytic acid, with binding energy − 7.1 kcal/mol, compared to that of *A. niger* and *P. pinophilum* (-6.7 kcal/mol), ensuring the stability of the interaction.

## Introduction

Phytic acid is a myo-inositol hexakisphosphate, the major storage form of phosphorus in plant tissues such as cereal grains, oilseeds, and legumes [1], by about 70% of the main ingredients of the animal feed [2]. The phosphorus form in the animal feed “phytate” is usually unavailable for the monogastric animals [3], due to the lack of phytase in their digestive tracts [4]. However, supplementation of the feed with phosphorus is quite costly, and usually associated with an environmental hazardous due to the excessive excretion of phosphorus by animals [5], causing a serious environmental pollution problem of phosphorus eutrophication, causing an algal blooms. In addition, phytate was considered as anti-nutritional agent by chelating the different metal ions, preventing their accessibility, create complexes with proteins, reducing the dietary phosphors bioavailability [6]. Phytic acid is the main storage form of phosphorus in plants, representing about 2–5% of weight of oilseeds, legumes, and grains, affecting the nutritional value of these foods by chelating the divalent/ trivalent metal cations, for its highly negative charge [7]. So, hydrolysis of phytate into myo-inositol and phosphorus using microbial phytases is one of the most sophisticated approaches to increase the nutritional values of the animal feed, improving the protein digestibility and minerals accessibility [8].

Phytase (EC 3.1.3.26) is an unique class of phosphor-monoesterases that hydrolyze phytate into myo-inositol and inorganic phosphate [9]. Phytases were produced from various plants, animals and microbial sources [8]. Based on the catalytic function and structure, phytases from fungi have been classified as histidine acid phosphatases [5]. Several fungal genera namely *Aspergillus, Penicillium, Myceliophthora, Mucor, Rhizopus* and *Trichoderma* were reported as potential phytase producers [5–8]. The feasibility of fungal growth under solid state fermentation for phytase production, than bacteria, has been commonly reported [10].

Solid state fermentation conditions for fungal growth on agricultural wastes, natural residues as substrates under low moisture content is one of the most common approaches for commercial production of phytases [2, 3, 11, 12]. Enzymes can be easily extracted, less time-consuming, with an economical affordability [12, 13]. Some solid substrates were generally used for phytase production such as citrus peels, wheat bran, wheat straw, soybean meal, rice bran, oil cakes, corn cobs, corn bran, and coconut oil cakes [14, 15]. Phytases gained a great interest in the fields of nutrition, environmental conservation, and biotechnology, from the stockholders over the past 20 years, for its profound role in improving the nutritional value of the plant material in the animal feed by liberating phosphate [10]. However, the availability, structural stability and catalytic efficiency of this enzyme are the challenges that limit their further commercial applications.

*Catharanthus roseus* is one of the most common medicinal plants of diverse pharmaceutical applications due to their richness with numerous bioactive alkaloids and terpenoids [15]. *Catharanthus roseus* has been reported as repertoire for endophytic fungi with the metabolic potency for production of bioactive metabolites and enzymes [15]. Thus, the objective of this work was to recover a novel fungal isolate from *C. roseus* with a conceivable ability to produce phytase with a robust catalytic efficiency and stability to hydrolyze phytic acid in various agroindustrial byproducts.

## Materials and methods

### Screening for phytases producing endophytic fungi

The samples of *Catharanthus roseus* were collected from the botanical garden of Zagazig University, Zagazig, Egypt, at October/2022, according to the standard guidelines, and used the source of endophytic fungi. The plant sample was identified by Dr. Marwa El-Demerdash, a plant Taxonomist, and the plant sample was deposited at the Herbarium of Botany and Microbiology Department, Faculty of Science, Zagazig University under deposition number HBM-125. The collected plant parts were surface sterilized by 70% ethanol for 2 min, and 2.5% sodium hypochlorite for 4 min, then washed with sterile distilled water [16, 17]. The plant leaves were segmented, placed on the surface of Potato Dextrose Agar (PDA) agar [18–20], and the plates were incubated for 15 days at 30 °C. The developed fungal hyphal tips were purified by subculturing on PDA media, and the purified fungal isolates were stored as a slope cultures at 4 °C. The fungal isolates were identified based on their morphological features [21, 22].

### Fermentation medium and culture conditions

Several agro-industrial byproducts namely wheat bran, peanuts peels, soybean peels, barley bran, corn husk, and dried chicory leaves were utilized as substrates for phytase production by the recovered fungal isolates. Five grams of each dried substrate were placed into 250 ml Erlenmeyer conical flasks, then moistened with 10 ml of salt solution of 0.5% glucose, 0.3 % NaNO_3_, 0.1% KH_2_PO_4_, 0.05% KCl, 0.05 % MgSO_4_.7H_2_O, dissolved in tap water [23, 24]. The flasks were autoclaved, inoculated with 5 ml of the fungal spore suspension, and the cultures were incubated at 30 °C for 8 day in static incubator. The enzyme was extracted by 50 ml potassium phosphate buffer (pH 7.5), agitation for 30 min at 200 rpm, filtration by Whatman No. 1 filter paper and clarified by centrifugation at 1000 rpm for 10 min, and the supernatant as the source of crude enzyme was kept at 4 °C.

### Phytase assay

The activity of phytase was assessed based on the released inorganic phosphate [25] from phytic acid as substrate. The reaction mixture contains 0.5 ml of phytic acid solution in potassium phosphate buffer (pH 7.4, 0.1 mM) and 0.5 ml of the enzyme preparation, incubated for 15 min at 40 °C. The enzymatic activity was stopped by 1% TCA, followed by adding of 0.5 ml of ammonium molybdate-sulfuric acid reagent (8 g ammonium molybdate, 5 g ferrous sulphate and 27 ml sulfuric acid in 100 ml distilled water). The developed ammonium molybdate-phosphate complex was measured after 5 min at 660 nm [26]. Authentic concentrations of the KH_2_PO_4_ were prepared (10–100 μmol), the ammonium molybdate-sulfuric acid reagent was used, and the absorbance was measured under the standard conditions. Blank medium without fungal inocula were used for zeroing the reaction. Blank of the substrate (phytic acid) dissolved in potassium phosphate buffer (pH 7.4, 0.1 mM) was used for zeroing the spectrophotometer. Technical triplicates for each assay for the biological triplicates were used. The concentration of inorganic ammonium molybdate-phosphate complex was determined from the fitting of the linear equation. One unit of phytase was expressed by the amount of enzyme releasing 1 μmole of inorganic phosphorus by min per mg enzyme concentration.

### Protein concentration

The protein content of the crude enzyme preparation was assessed by Folin reagent [27], using bovine serum albumin as a standard.

### Identification of the most potent phytase producing fungal isolates

The most potent phytase-producing fungal isolates were identified based on their morphological features according to the reference keys [21, 22]. The morphologically identified fungal isolate were molecularly confirmed based on the sequence of their internal transcribed spacer (ITS) [28, 29]. The fungal mycelia (0.1 g) were minced in liquid nitrogen, and dispensed in 0.5 ml CTAB extraction buffer (2% CTAB, 2% PVP40, 0.2% 2-mercaptoethanol, 20 mM EDTA, 1.4 mM NaCl in 100 mM Tris-HCl, pH 8.0). The genomic fungal was extracted by phenol-chloroform-isoamyl alcohol [30], used as a template for PCR with the primer sets ITS4 5′-GGAAGTAAA-AGTCGTAACAAGG-3′ and ITS5 5′-TCCTCCGCTTATTGATATGC-3′ [17, 31]. The PCR reaction had 10 μl 2 × PCR master mixtures (Cat. # 25027), gDNA, primers (10 pmol/μl), in 20 μl distilled water, according to the manufacturer’s instructions. The PCR was programmed as initial denaturation 94 °C for 4 min, 35 cycles by denaturation at 94 °C for 30s, annealing at 53 °C for 20 s, extension at 72 °C for 40 s, and final extension at 72 °C for 5 min. The amplicons were analyzed by 1.5 % agarose gel, sequenced by Applied Biosystems, HiSQV Bases with the same primers. The sequence was non-redundantly BLAST searched on the NCBI database, aligned with Clustal W, and the phylogenetic tree was constructed with neighbor-joining method of MEGA X [32].

### Maximizing the productivity of phytases by *A. terreus* under solid state fermentation by the response surface methodology

The physicochemical requirements for phytase production by *A. fumigatus* were optimized by the response surface methodology under solid state fermentation [19, 33–35]. The nutritional parameters such as sucrose, fructose, maltose, glucose, lactose, peptone, ammonium chloride, sodium nitrate, yeast extract, methionine, glycine, asparagine, glutamine, peptone, pH at different incubation time were screened for phytase production by *A. terreus* by the Plackett-Burman Design. The variables were symbolized by high (+1) and low (−1) levels. The most significant variables (sucrose, sodium nitrate, yeast extract and temperature) affecting phytase productivity were optimized by the face-centered central composite design (FCCD), to determine the mutual interactions of the variables. The variables were represented by low (−1), medium (0), and high (+1), and the center point was repeated 5 times.

### Purification and molecular subunit structure of the purified enzyme

The potent phytase-producing fungal isolate was grown on the optimized medium under solid state fermentation conditions, incubated at the desired conditions, the cultures were filtered, the enzyme extracted by potassium phosphate buffer as described above. The crude protein of the cultural filtrate was precipitated and purified by the committed purification protocols [28–30, 36–40]. The crude proteins was fractionally concentrated by 20 kDa cut-off dialyzer (Cat.# 546–00051) against polyethylene glycol 6000, followed by 30-kDa Ultracentrifugal membrane (Amicon, Millipore). The enzyme was purified by gel-filtration with Sephadex G_200_ column, and the fractions were eluted in Tris-HCl buffer (pH 7.4, 0.1 mM), and the enzyme activity and concentrations were determined as above. The most active fractions were gathered and concentrated by 30-kDa Ultracentrifuge membrane, prior to the further purification step. The enzyme was purified by ion-exchange chromatography with Sepharose 4B column, eluted by Tris-HCl buffer (pH 7.4, 0.1 mM) with a gradient NaCl concentrations (0, 100, 200 and 300 mM), and the activity and protein concentrations of the enzyme of each fraction were assessed as mentioned above. The most active fractions were selected, and the molecular homogeneity and subunit structure of the purified enzyme was checked by the SDS-PAGE [41], compared to authentic protein marker (Puregene, Cat.# PG-PMT2962, 315–10 kDa).

### Biochemical properties of the purified phytase from *A. terreus*

The biochemical properties of the purified enzyme including reaction temperature, reaction pH, pH stability, thermal stability, response to inhibitors/activators and substrate affinity were assessed according to our previous studies [28, 29, 36–44]. The effect of reaction pH (5–9) on phytase activity was assessed by 0.1 mM of citrate-phosphate buffer and Tris-HCl. The pH stability was evaluated by pre-incubating the enzymes at different pHs (5.0–9.0) for 2 h at 4 °C, then measuring their residual activity by the standard assay. The effect of various inhibitors mainly cations Cu^2+^, Mg^2+^, Fe^3+^, Hg^2+^, Fe^3+^, Ba^2+^, Zn^2+^ and Ca^2+^ in addition to benzoic acid and hydroxylamine on the activity of purified enzyme by incubating at 1 mM, for 2 h at 4 °C, then measuring the residual enzymatic activities. The thermal stability of the purified enzyme was assessed by pre-incubating without substrate at 20, 40, and 50 °C, for different time points “till 200 min” then measuring the enzyme activity by the standard assay.

### Applications of the purified phytase in wheat bran as animal feed

Wheat bran as a component of the animal feeds has a significant amount of phytate as estimated by about 5% [45]. The toxicity of the purified *A. terreus* phytase has been checked by the brine shrimp assay, and the enzyme has no any signs of toxicity as reveled from the mortality rate of brine shrimp larvae [42]. The effect of the purified enzyme to hydrolyze phytic acid in wheat bran was assessed, by amending the enzyme to the wheat bran at 5: 1 v/w, incubating for 2 h at 50 °C [46]. After the incubation of the solid state fermented cultures, the residual phytate was extracted [46, 47], with minor modifications. Breifly, 5 grams of the enzyme-treated wheat bran were extracted with 50 ml 0.2 N HCl for 3 h with a magnetic stirrer at room temperature, the extracts was centrifuged at 5000 rpm g for 4 min. The supernanat (5 ml) was mixed with iron III-thiocyanate reagent (0.1 g iron(III), 0.5 g ammonium thiocyanate and 0.2 ml of 5 N HNO_3_ in 100 ml distilled water) [48]. The mixture was stirred in water bath at 40 °C for 2 h then cooled at room temperature, centrifuged 5 min at 5000 rpm, and the phytic acid concentration was assessed by HPLC (Chen et al., 2025). The HPLC system (YOUNG In, Chromass) was used with reverse-phase C18 column (Cat.# 959963–902) with mobile phase of 30% acetonitrile and 0.1 M HNO_3_ at flow rate 1 ml/min [48]. The samples (20 μl) were injected into the HPLC column, the absorbance was measured at wavelength of 460 nm for 20 min retention time. Authentic concentrations of phytic acid (10–100 mg/ml) were used under the same conditions, and the phytic acid concentration was determined.

### Molecular docking analysis

The molecular docking analysis to elucidate the predicted interactions of *A. terreus* phytase and phytic acid as substrate was conducted. The active sites, binding affinity, and the visual representations of the docking interactions were shown by the PyMOL, Discovery Studio, and Chemira X. Avogadro software package was used for preparing phytic acid molecules before docking simulations [49]. The sequences of phytase from *A. terreus, A. niger* and *Penicillium* sp were obtained from NCBI. The molecular Docking tools PyRx [50] and AutoDock Vina [51] were used to simulate the interaction between phytase and phytic acid. The docking results were analyzed using PyMOL and Discovery Studio [51] to determine the binding affinity and to visualize the binding sites involved in the interaction between the enzyme and substrate.

### Fungal deposition

The ITS sequence of *Aspergillus terreus* EFBL-AS has been deposited to the GenBank with accession # PV412881.1.

### Statistical analysis

The experiments were performed in triplicates, and the results were expressed by mean ± STDV. The statistical analysis was assessed using one-way ANOVA (SPSS software v.18) test, and the means were compared with Duncan’s test at 0.05 level.

## Results and discussion

### Isolation of the endophytes of *Catharanthus roseus*: Screening for phytase production under solid state fermentation

**Fig. 1:**
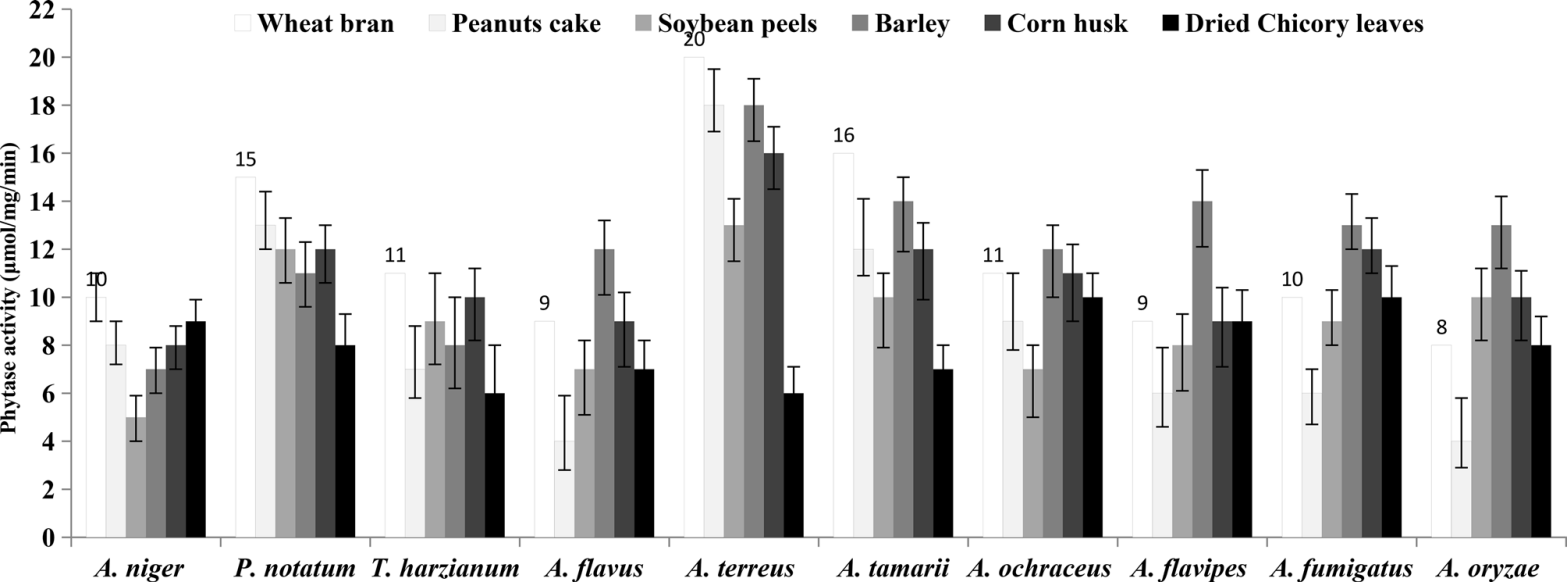
Screening for phytase production from the endophytic fungi of *Catharanthus roseus*, grown on different substrates under solid state fermentation bioprocess

Ten endophytic fungal isolates inhabiting *Catharanthus roseus* were isolated on PDA medium. These fungal isolates were morphologically identified to the their species levels as *Aspergillus niger, A. flavus, A. fumigatus, A. ochraceous, A. flavipes, A. terreus, A. tamarii* and *A. oryzae,* in addition to *Penicillium notatum* and *Trichoderma harzianum.* Solid state fermentation being one of the most economically affordable cultural conditions especially for fungi for their lower moisture contents, that favor the germination of the fungal spores [42, 52]. These fungal isolates were grown on different solid substrates as wheat bran, peanuts cake, Soybean peels, Barely bran, Corn husk, and dried chicory plants, at 5% initial moisture contents for 10 days. After incubation, the enzyme was extracted, and their activity and concentration were assessed as described in Materials and Methods. From the obtained results (Fig. 1), the highest phytase productivity was reported for *A. terreus* grown on wheat bran (20 μmol/mg/min), followed by peanut cake and soybean peels (18 μmol/mg/min) under solid state fermentation. While, the phytase yield by the other fungal isolates “*A. niger, A. flavus, T. harzianum, A. ocharceous, A. flavipes* and *A. fumigatus”* was ranged between 9–12 μmol/mg/min. *Catharanthus roseus* has been reported as a source of a plethora endophytic fungi with diverse endophytic fungi [19, 33]. The fluctuation on the phytase productivity of the same fungal isolate with the solid substrates, ensures effect of the chemical constituents of this substrate especially for phytic acid on inducing phytase production. The highest yield of phytase by *A. terreus* on wheat bran, could be attributed to the highest content of phytic acid that reported by about 4–5% [45, 53], compared to the phytic acid content of peanut cake that about 1.36% [54], unlike to the low phytic acid yield in corn husk [55]. Thus, the affordability of wheat bran as favorable substrate for phytase production could be due to their higher contents of phytic acid. The highest productivity of phytase by *A. terreus*, could be due to the versatility of this fungus for production of panel of cellulosic, and ligninolytic enzymes, facilitating the rapid assimilation of wheat bran as substrate under solid state fermentation conditions. The water activity (aw) required for the fungal growth is about 0.5–0.6, compared to the bacterial requirement that requires 0.8–0.9, making the solid state conditions much more feasible for the fungal growth and physiological process [56]. Unlike submerged conditions, the solid state fermentation bioprocess being more attractive for the higher affordability for fungal growth, higher metabolic productivity, low water contents that the reduced risk of bacterial contamination, lower energy requirements [52].

### Identification of the potent fungal isolate

**Fig. 2:**
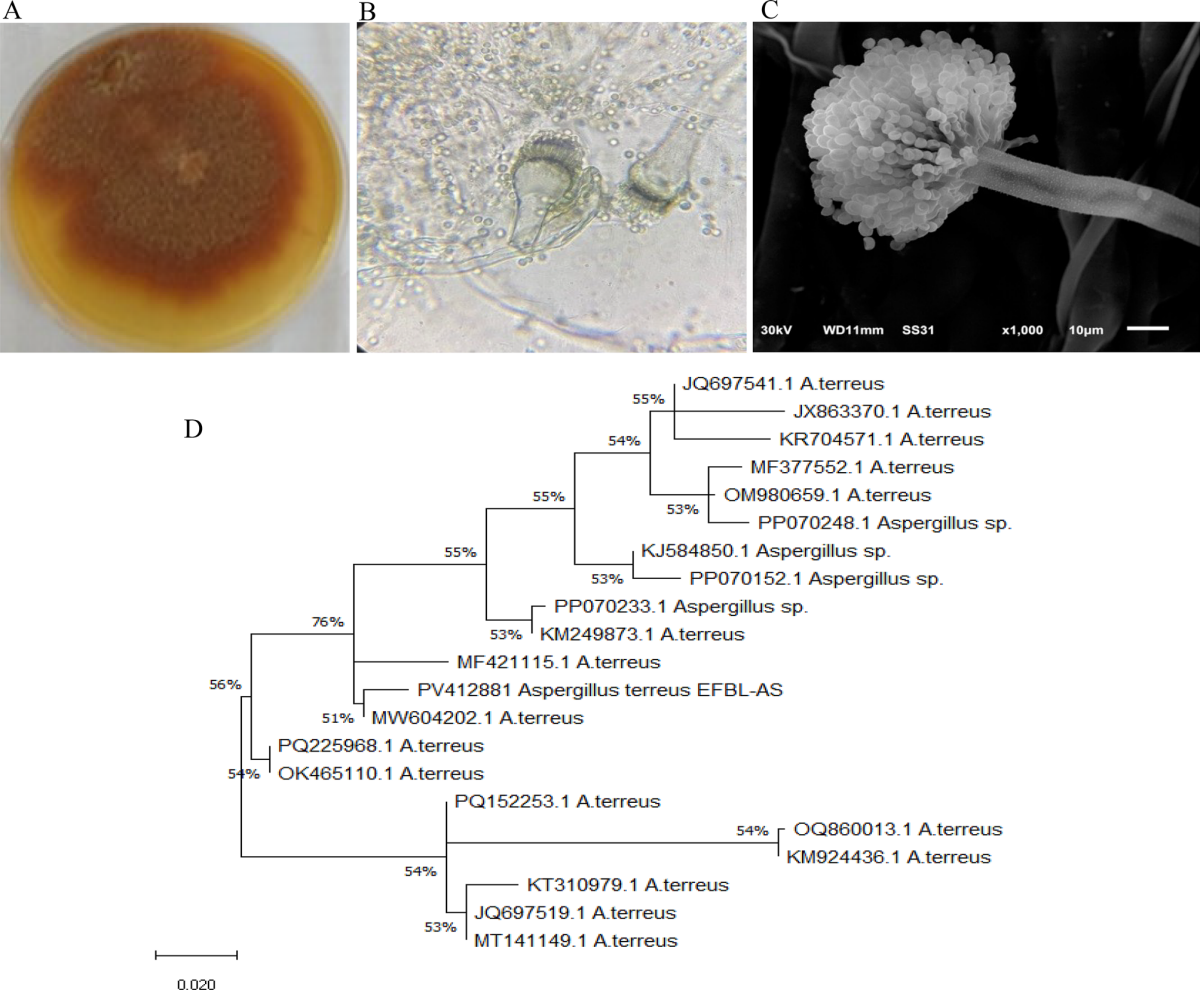
Morphological and molecular identification of the most potent phytase-producing fungal isolate inhabiting *C. roseus.* The plate culture (**A**) and conidial head of *A. terreus* at 100 X (**B**) and at 1000 X (**C**). The PCR amplicons of the ITS region of *A. terreus* was sequenced and BLAST searched on the NCB. The molecular phylogenetic analysis of the ITS sequence of *A. terreus* was conducted by the Maximum Likelihood method with MEGA X software package (**D**).

The experimented fungal isolate had a colony color ranged from cinnamon to orange-brown, long, compact, columnar and biseriate conidial heads (Fig. 2) that typically follows the morphological traits of *A. terreus* [21, 22]. The identity of the most potent phytase-producing fungal isolate was molecularly confirmed based on their ITS sequence. The PCR of the ITS amplicon about 650 bp (Fig. 2), was purified, sequenced, and non-redundantly BLAST searched on the NCBI database. The ITS sequence of the current isolate had a 99.73% similarity, and zero E-value with the ITS sequence of *A. terreus* MW604202.1, MF421115.1, KM249873.1, PP070233.1, PP070152.1, KJ584850.1, PP070248.1, OM980659.1, and MF377552.1. The ITS sequence of *A. terreus* EFBL-AS was deposited into the Genbank with accession # PV412881.1. Thus, from the morphological description and molecular verification, the isolate was identified as *A. terreus,* class Ascomycota, subclass; Pezizomycotina; Eurotiomycetes; Eurotiomycetidae; Eurotiales; Aspergillaceae; *Aspergillus; Aspergillus* subgenus Circumdati.

### Nutritional bioprocess optimization of *A. terreus* for maximizing the phytase productivity

**Fig. 3:**
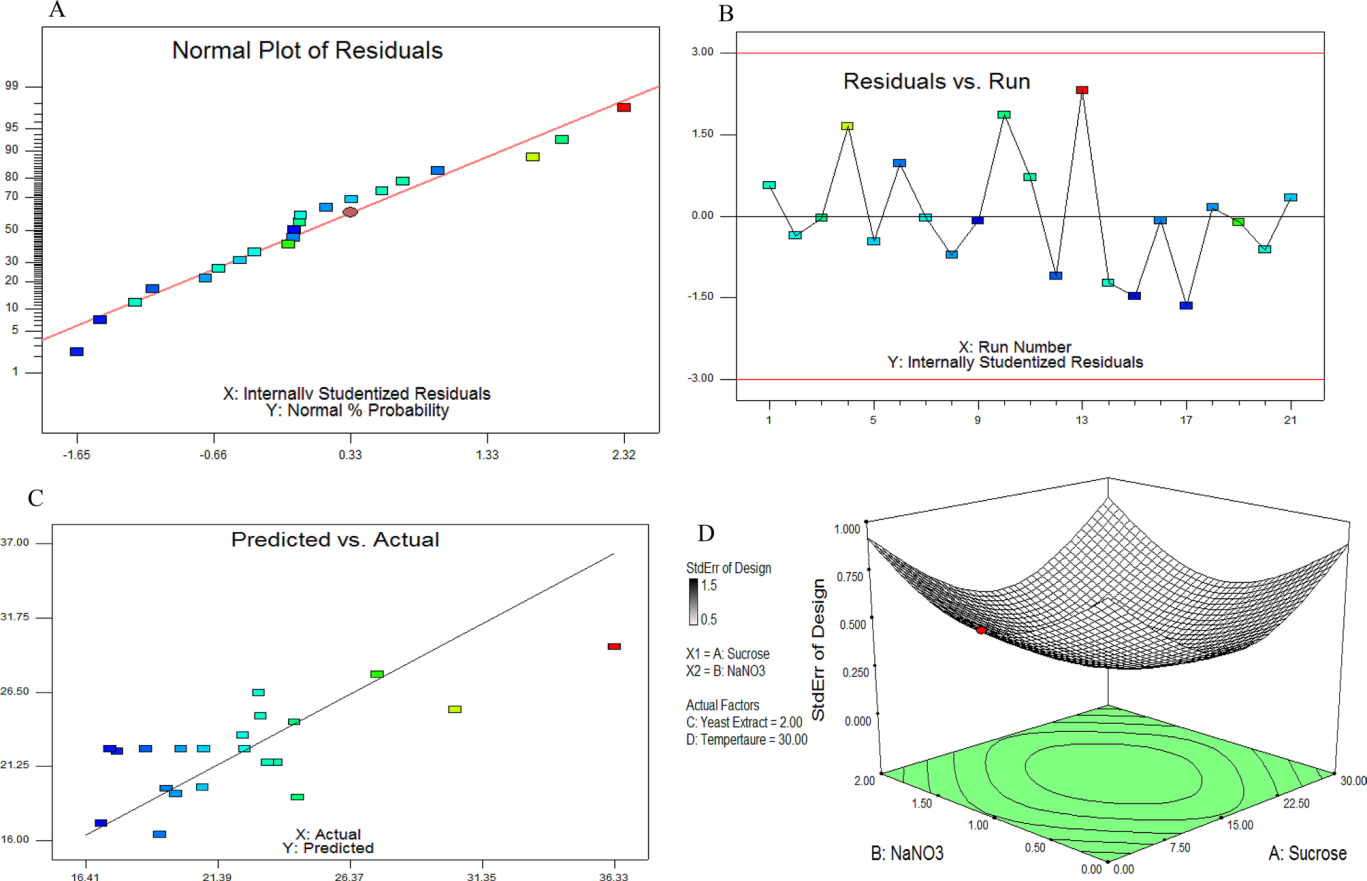
The main effects of the different variables on phytase production by *A. terreus* with the FCCD. The normal plot of the residual (**A**), plot of residuals versus runs (**B**), and plot of residual versus actual phytase productivity (**C**). The three-dimensional surface plots for interactions of the NaNO3 and sucrose variables on phytase production by *A. terreus* by the CCD (**D**).

The productivity of phytase by *A. terreus* was optimized by growing on wheat bran under solid state fermentation, as the affordable substrate supporting the maximum phytase productivity, as reported above. Phytase productivity by *A. terreus* grown on wheat bran was optimized by the Faced Central Composite Design (CCD) of the response surface methodology [19, 34]. The wheat bran was moistened with 5.0 % salt solution with different concentrations of sucrose, NaNO_3_, yeast extract, incubated at different temperatures as shown on the matrix of FCCD, at five levels (Table 1). The interactions of the tested parameters were assessed from the FCCD. From the FCCD matrix (Table 1), the highest actual and predicted phytase productivity by *A. terreus* was obtained at run # 13 with 36.3 μmol/mg/min and 33.3 μmol/mg/min, respectively, with residual 3.0. At run #13, the experimented parameters were NaNO_3_ (0.2 %), yeast extract (0.4 %) at incubation temperature 35 °C. From the CCD results, the actual productivity of phytase ranged from 17.0 to 36.3 μmol/mg/min, ensures the significant of the design. So, upon FCCD nutritional bioprocessing, the yield of phytase was increased by approximately 1.5 folds compared to control cultures of *A. terreus* (20 μmol/mg/min), grown on wheat bran under solid state fermentation. The different plots of the FCCD analyses of bioprocessing of phytase by *A. terreus* such as normal plots of residuals, plot of residual with the runs, plots of predicted with the actual yield of phytase, in addition to the 3D view of tested parameters were shown in Fig. 3. From the statistical analysis by ANOVA of the FCCD optimization bioprocess, the F-value was 4.24 and *p*-value 0.0158, that ensure the significance of the designed model (Table 2). The Model F-value of 4.24 implies the model is significant. Values of “Prob > F” less than 0.0500 indicate model terms are significant. So, temperature is most significant parameters, followed by sucrose. From the CCD results, the phytase productivity was expressed by the formula:

$$\eqalign{& Phytase\,productivity\,\left( {\mu mol/mg/min} \right)\, \cr& = \,3.41415 - 0.1258 * Sucrose\, + \,0.56619\,*\,NaN{O_3}\, \cr& + \,0.8157\,*\,Yeast\,Extract\, + \,0.62548\,*\,Temperature \cr} $$

**Table 1:** Matrix of the Faced Central Composite Design (CCD) for phytase production A. terreus grown on wheat bran under solid state fermentation

Run	Sucrose (g/l)	NaNO_3_ (g/l)	Yeast Extract (g/l)	Temperature (°C)	Actual activity (μmol/mg/min)	Predicted (μmol/mg/min)	Residual
1	15	0	2	30	23.2	21.4	1.8
2	15	2.6	2	30	22.3	23.4	−1.1
3	0	1	2	30	24.6	24.3	−0.03
4	15	1	5	30	30.3	25.3	5.03
5	15	1	2	30	20.8	22.5	−1.63
6	30	2	0	25	19.2	16.5	2.7
7	15	1	2	30	22.4	22.5	−0.1
8	15	1	2	30	20	22.5	−2.5
9	15	1	2	21	17	17.2	−0.2
10	0	0	0	25	24.4	19.1	5.3
11	30	0	0	35	23.6	21.5	2.1
12	15	1	2	30	18.6	22.5	−3.8
**13**	**0**	**2**	**4**	**35**	**36.3**	**33.3**	**3.0**
14	0	2	0	35	22.9	26.4	−3.4
15	15	1	2	30	17.3	22.5	−5.1
16	30	2	4	25	19.4	20	−0.55
17	0	0	4	25	17.6	22.3	−4.7
18	40	1	2	30	19.8	19.4	0.4
19	15	1	2	38	27.4	27.7	−0.3
20	30	0	4	35	23	24	−1
21	15	1	0	30	20.8	19.4	1.4

**Table 2:** ANOVA analysis of the CCD optimization process of phytase by A. terreus under solid state fermentation process

Source	Sum of Squares	df	Mean Square	F-Value	p-value Prob > F		
Model	216.34	4	54.08	4.24	0.0158	significant	
A-Sucrose	42.04	1	42.04	3.29	0.0884		
B-NaNO_3_	4.38	1	4.38	0.34	0.5663		
C-Yeast Extract	36.35	1	36.35	2.85	0.1109		
D-Temperature	133.57	1	133.57	10.46	0.0052		
AB	6331.21	1	33,317.1	3.45	0.084		
AC	215.2	1	215.5	0.022	0.87		
AD	1401.79	1	2402.9	0.25	0.58		
BC	1956.4	1	1953.4	0.2	0.61		
BD	118.07	1	128.7	0.009	0.964		
CD	7787.5	1	6787.5	0.7	0.278		
A^2	9679.5	1	9679.5	1.0	0.265		
B^2	12,839.5	1	22,831.6	3.17	0.185		
C^2	13,760.7	1	13,760.5	2.14	0.199		
D^2	81,361.46	1	91,361.47	7.8	0.012		
Residual	202.26	16	12.77				
Lack of Fit	128.97	12	15.75	4.12	0.0914	not significant	
Pure Error	15.29	4	3.82				
Cor Total	420.6	20					

The Model F-value of 4.24 implies the model is significant. There is only a 1.58% chance that a “Model F-Value” this large could occur due to noise. Values of “Prob > F” less than 0.0500 indicate model terms are significant. In this case D are significant model terms. Values greater than 0.1000 indicate the model terms are not significant. The “Lack of Fit F-value” of 4.12 implies there is a 9.14% chance that a “Lack of Fit F-value” this large could occur due to noise. Lack of fit is bad– we want the model to fit

This relatively low probability (<10%) is troubling

From the CCD analysis, the maximum productivity of phytase was resolved at run #13 in absence of sucrose, ensuring the preference of wheat bran to be utilized as carbon source for the enzyme production, since wheat bran contains about 13% fibers and 4% oligosaccharides [53]. The inhibition of phytase productivity by *A. terreus* grown on wheat bran, could be due to the glucose effect, that triggers the expression of specific transcriptional factors, thus, inhibiting the expression of the phytase encoding genes. Filamentous fungi can produce abundant carbohydrate hydrolyzing enzymes with high activity, greater potential to decompose plant cell-wall polysaccharides [57].

### Purification and molecular subunit structure of *A. terreus* phytase

**Fig. 4:**
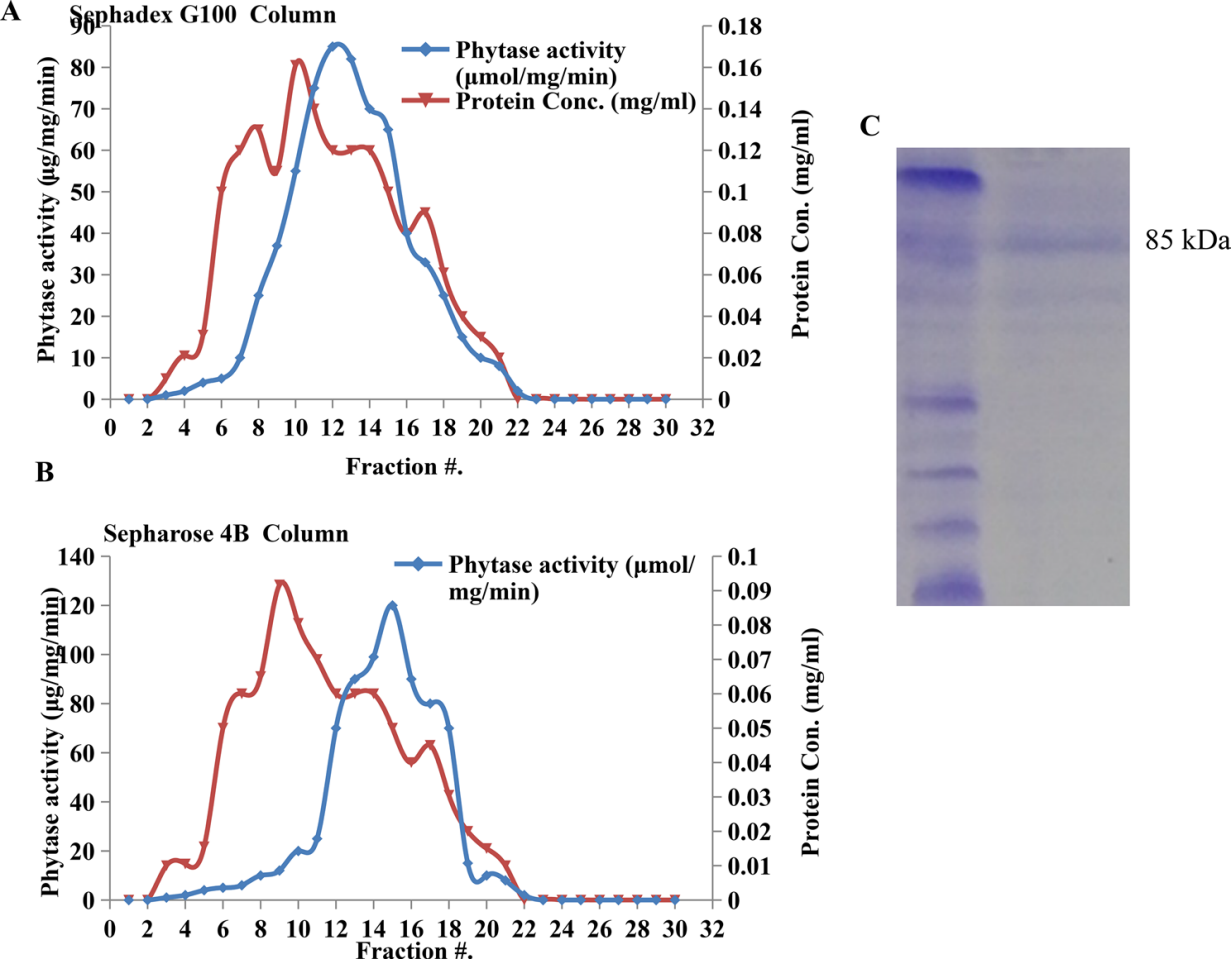
Purification of phytase from *A. terreus* grown on wheat bran under solid state fermentation. After incubation of the cultures, the crude proteins were extracted and precipitated by acetone. The enzyme was purified by gel-filtration using Sephadex G100 (**A**) and ion-exchange chromatography by Sepharose 4B column (**B**). (**C**) The SDS-PAGE profile of the purified phytase from *A. terreus*

*Aspergillus terreus* was grown on the nutritionally optimized medium of wheat bran under solid state fermentation bioprocess as reported above. After cultural incubation, the enzyme was extracted by potassium phosphate buffer, purified by the committed purification protocol of gel-filtration and ion-change chromatography [43, 44, 58–61]. The crude proteins were precipitated with child acetone (1/1 v/v), the activity of phytase was increased into 50 μmol/mg/min, compared to the crude enzyme (36 μmol/mg/min). The overall purification profile of phytase from *A. niger* was summarized in Table 3. The precipitated proteins were fractionated by gel-filtration chromatography using Sephadex-G100 column, the activity and protein concentrations of the fractions were assayed. The most active fractions were combined, with a plausible activity 90 μmol/mg/min, purification folds 2.5 with 80.5 % yield compared to the crude enzyme (Fig. 4). The active fractions were further purified by ion-exchange chromatography using Sepharose 4B column, eluted with the gradient NaCl concentrations. The activity of phytase of *A. terreus* was increased into 125 μmol/mg/min, with 3.4 purification folds and overall yield 62.7%, compared to the crude enzyme (36 μmol/mg/min) (Fig. 4). The most active and molecular homogenous fractions were collected and concentrated by 30 kDa Amicon ultracentrifugal membrane. Similar purification protocols were reported for phytase purification from solid cultures of fungi [69].

**Table 3:** Purification profile of phytase from solid state fermented cultures of Aspergillus terreus grown on wheat bran as substrate

	Total activity (μmol/min)	Protein content (mg)	Specific activity (μmol/mg/min)	Yield (%)	Purification fold
Crude enzyme	55.86	1.55	36	100	1
Acetone precipitate	50	1.01	50	89.5	1.39
Sephadex G_100_	45	0.5	90	80.5	2.5
Sepharose 4B column	35	0.28	125	62.7	3.48

The enzyme molecular subunit structure was determined by SDS-PAGE. The subunit molecular mass of the purified phytase from *A. terreus* was resolved as a sharp band of 85 kDa as revealed from the SDS-PAGE. Consistently, the molecular subunit of phytase of *A. terreus* was consistent with those reported for the phytases from *Aspergillu*s spp [6, 8, 9, 11, 14]. However, there are reports that indicate the existence of phytases which exist as dimers and even tetramers.

### Biochemical properties of the purified phytase of *A. terreus*

**Fig. 5:**
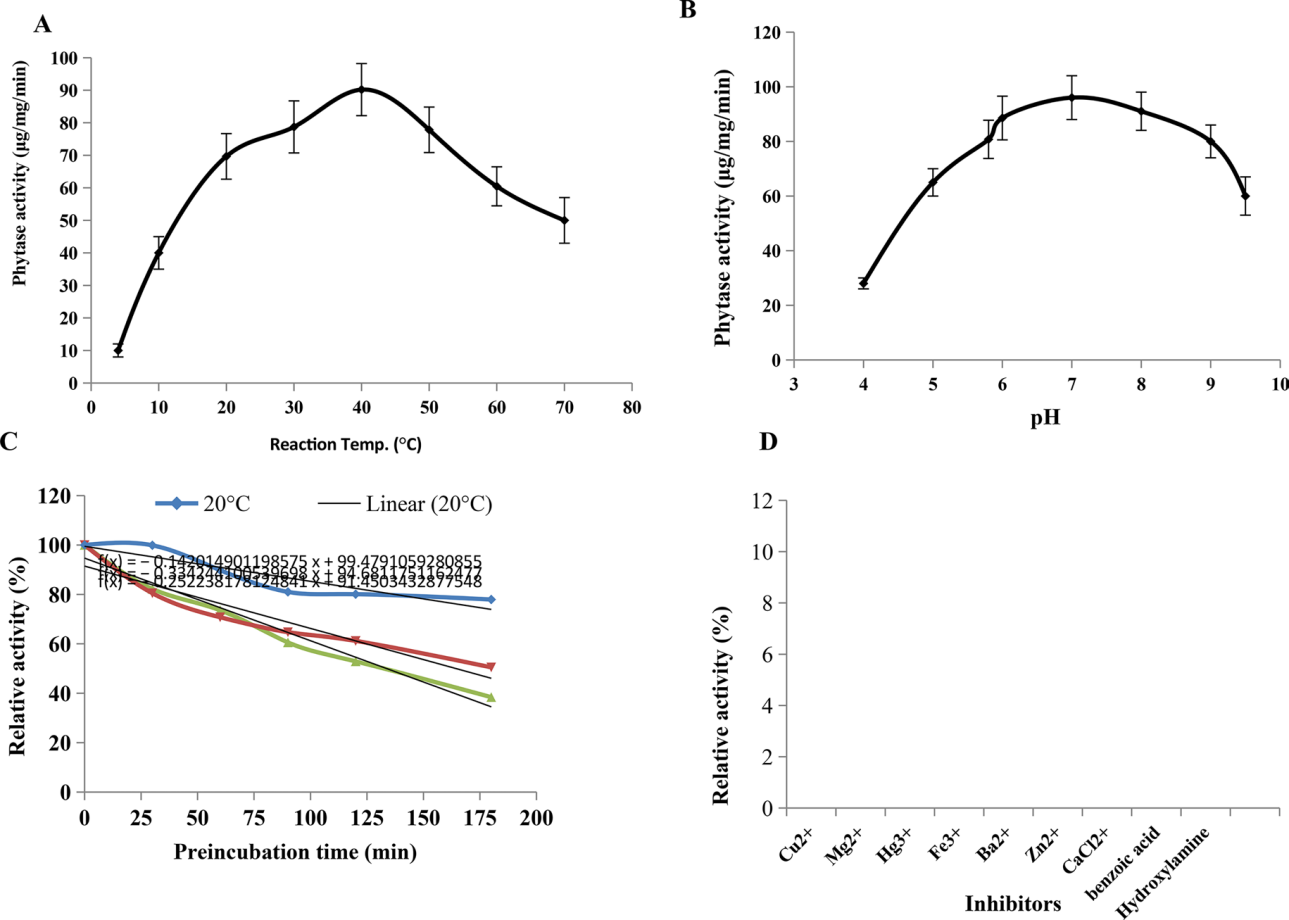
Biochemical properties of the purified phytase of *A. terreus* grown on wheat bran under solid state fermentation. The effect of reaction temperature (**A**), reaction pH (**B**), thermal stability (**C**) and metals ions and inhibitors (**D**) on the activity of phytase were assessed

The effect of reaction temperature on the activity of purified phytase of *A. terreus* was determined at reaction temperature from 10–70 °C, under the standard assay conditions. A substantial increase on the enzyme activity was observed with the reaction temperature, till maximum temperature 37–40 °C, followed by an obvious decrease to the enzyme activity. The maximum activity of *A. terreus* phytase was reported at 37–40 °C, followed by noticeable reduction to the enzyme activity at reaction temperature 70 °C. At 70 °C, the activity of phytase was reduced by about 1.8 folds, compared to 37–40 °C (Fig. 5). Similarly, the highest activity of purified phytase from *A. niger* [6, 14] was detected at 40 °C. The similar reaction temperature with the previously reported phytase from fungi, ensures their structural proximity with the enzyme from the different fungal sources. The enzyme thermal stability is one of the most crucial parameters affecting the potency of enzyme in the further industrial applications [9].

The effect of reaction pH (4–9) on the activity of *A. terreus* phytase was assessed. An obvious increase on the phytase activity was observed with the reaction pH, till maximum activity at pH 7.0 (96.1 μmol/mg/min), followed by decrease in the enzyme activity with higher pHs (pH 10) (Fig. 5). So, at pH 7.0, the activity of phytase was slightly increased by about 1.1 folds compared to the control at pH 6.5 (90.1 μmol/mg/min). Noticeably, a strong reduction to the phytase activity was reported at acidic pHs than that the alkaline one, ensure the higher denaturation of the enzyme tertiary structure at acidic pH. The significant decreasing on the enzymes activity at the acidic pHs was reported frequently for various enzymes derived from fungi [43, 44, 58, 59], that might be due to the identity of N-terminal sequence, charges, molecular subunit structure and assembly of subunits. The optimum reaction pH of *A. terreus* being higher than that reported for phytase from *A. niger, A. carneus* (5.0–5.4) [5, 14].

The thermal stability of the purified phytase of *A. terreus* was assessed by pre-incubating the enzyme at 20, 40, and 50 °C, then the residual enzymatic activity was measured by the standard assay. From the thermal stability profile, the activity of phytase was reduced with the higher temperature as revealed from the half-life time and thermal denaturation rate as summarized in Table 4. The half-life time of *A. terreus* phytase was 124.5, 10.8, 5.2 and 3.8 h, at 4 °C, 20 °C, 40 °C, and 50 °C, respectively. The thermal denaturation rate (Kr/min) was 0.009×10^−3^, 0.095 ×10^−3^, 0.27 × 10^−3^ and 0.292 × 10^−3^ at 4, 20, 40, and 50 °C, respectively. So, the thermal denaturation rate and half-life time were reduced sequentially with the pre-incubation temperature. The half-life temperature of the purified phytase of *A. terreus* was estimated to be 111.5 °C, so, the enzyme loss 50% of its initial activity by heating at 111.5 °C for 60 min. So, phytase of *A. terreus* had a higher thermal stability than the enzyme of *A. fumigatus* and *A. ficuum* [5, 8]. The activity of *A. niger* phytase was reduced by about 78% by thermal treatment at 80 °C for 3 min [5]. So, the robust thermal stability of *A. terreus* phytase gave this enzyme a feasibility privilege in food and feed industries, by improving their nutritional values and mineral bioavailability. Practically, integration of phytase with a conceivable thermal stability, in addition to chemical modification via different immobilization approaches to fortify the enzyme structure and thermal structural/ catalytic stability is one of the pivotal approach to intensify the industrial potential of this enzyme as reviewed by Venkataraman et al. [31]. The influence of various metal ions on the catalytic identity of purified phytase of *A. terreus* was assessed. The enzyme was demetallized by dialysis against 1.0 mM EDTA to resolve the apo-enzyme. From the results (Fig. 5), there is no obvious effect by the different metal ions on the enzyme activity, except the slight negative effect by Ca^2+^ ions. So, the obvious resistance/ independence of the enzyme catalytic activity on metals ions, ensures the feasibility of using this enzyme in the food and feed products for hydrolysis of phytate. From the enzyme activity, there is no obvious significant variations on the activity purified phytase for the tested metal ions except the slight negative effect by the Ca^2+^ ions. Similar results, were reported for the inhibitory effect of Ca^2+^ ions on phytase acativity from *A. niger*, and *Rhizopus* sp [5, 14].

**Table 4:** Thermal kinetic parameters of the purified A. terreus phytase

Temperature (°C)	T_1/2_ (h)	Kr (min) x10^−3^	Tm (°C)
4	124.5	0.009	111.5
20	10.8	0.095	
40	5.2	0.27	
50	3.8	0.292	

The affinity of the purified phytase of *A. terreus* towards the phytic acid, fructose-1,6 diphosphate and ATP was assessed. From the kinetic parameters (Table 5), the highest affinity (*K*_*m*_ value) of *A. terreus* phytase was reported for phytic acid (0.025 mM) followed by ATP (0.19 mM) and Fructose 1,6 diphosphate (0.28 mM). The highest catalytic efficiency by *A. terreus* phytase for phytic acid, ATP and fructose 1,6-diphosphate was 2040, 242.2 and 145.5 mM^−1^s^−1^, respectively. Similar kintics properties were reported for phytase from *Aspergillus ficuum* [19], *A. fumigatus* [20], *A. japonicus* [29].

**Table 5:** Kinetics of A. flavus GST activity towards various substrates

	Vmax (μmol/mg/min)	Km (mM)	Kcat (s-^1^)	Kcat/Km (mM^−1^s^−1^)
Phytic acid	125	0.025	51	2040
Fructose 1,6-diphosphate	92	0.28	38	135.5
ATP	102	0.19	46	242.2

### Application of phytase of *A. terreus* in hydrolyzing of phytic acid in wheat bran

**Fig. 6:**
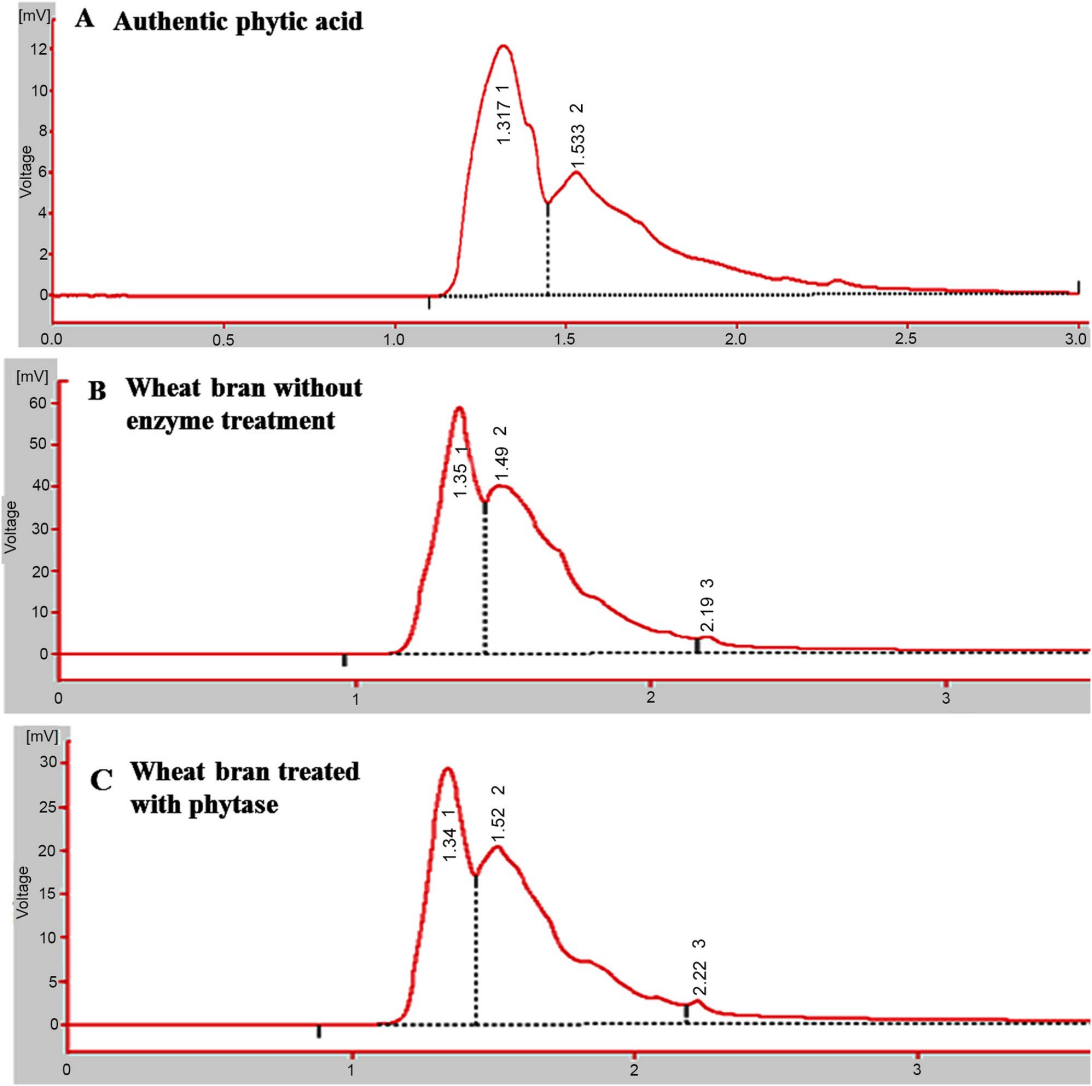
Phytic acid contents of wheat bran in response to the purified phytase of *A. terreus.* The wheat bran was treated with the purified phytase of *A. terreus*, then phytic acid was extracted and determined by HPLC. The HPLC chromatogram of the authentic phytic acid (10 μg/ml) (**A**), wheat bran-phytic acid without (**B**) and with phytase treatment (**C**)

The activity of purified phytase for hydrolyzing phytic acid in wheat bran was assessed. Wheat bran was amended with the enzyme (1:5 w/v), for 2 h at 40 °C, then phytic acid was extracted and determined by HPLC. From the HPLC profile (Fig. 6), the initial concentration of phytic acid of wheat bran (50 mg/g) was reduced into 8 mg/g in response to phytase treatment, i.e 6.5 folds reduction upon enzyme treatment. As revealed from the peak area, the concentration of phytic acid in wheat bran was reduced by 85 %, upon using *A. terreus* phytase compared to the untreated wheat bran. The activity of purified *A. terreus* phytase of hydrolyzing phytic acid in wheat bran ensures the feasibility of this enzyme as an animal feed additive to increase the nutritional values of feed by converting phytic acid to inositol and phosphate, thus improving the phosphorus availability and nutrient utilization, enhancing the mineral bioavailability and nutrient assimilation [62–65]. Phytic acid is the main anti-nutrient agent by preventing the absorption of protein, divalent and trivalent metal ions (Fe^2+^, Fe^3+^, Ca^2+^, Mg^2+^, Zn^2+^, Cu^2+^) from the diet, due its negative charge identity [16–18]. Monogastric animals, like pigs, poultry, and fish, have no or low amounts of gastrointestinal phytases, preventing them from using the phytate, phosphorus found in food and livestock feed. As a result, they require inorganic phosphate supplementation to satisfy their dietary and growth requirements, which raises feed costs and phosphorus pollution levels.

**Fig. 7:**
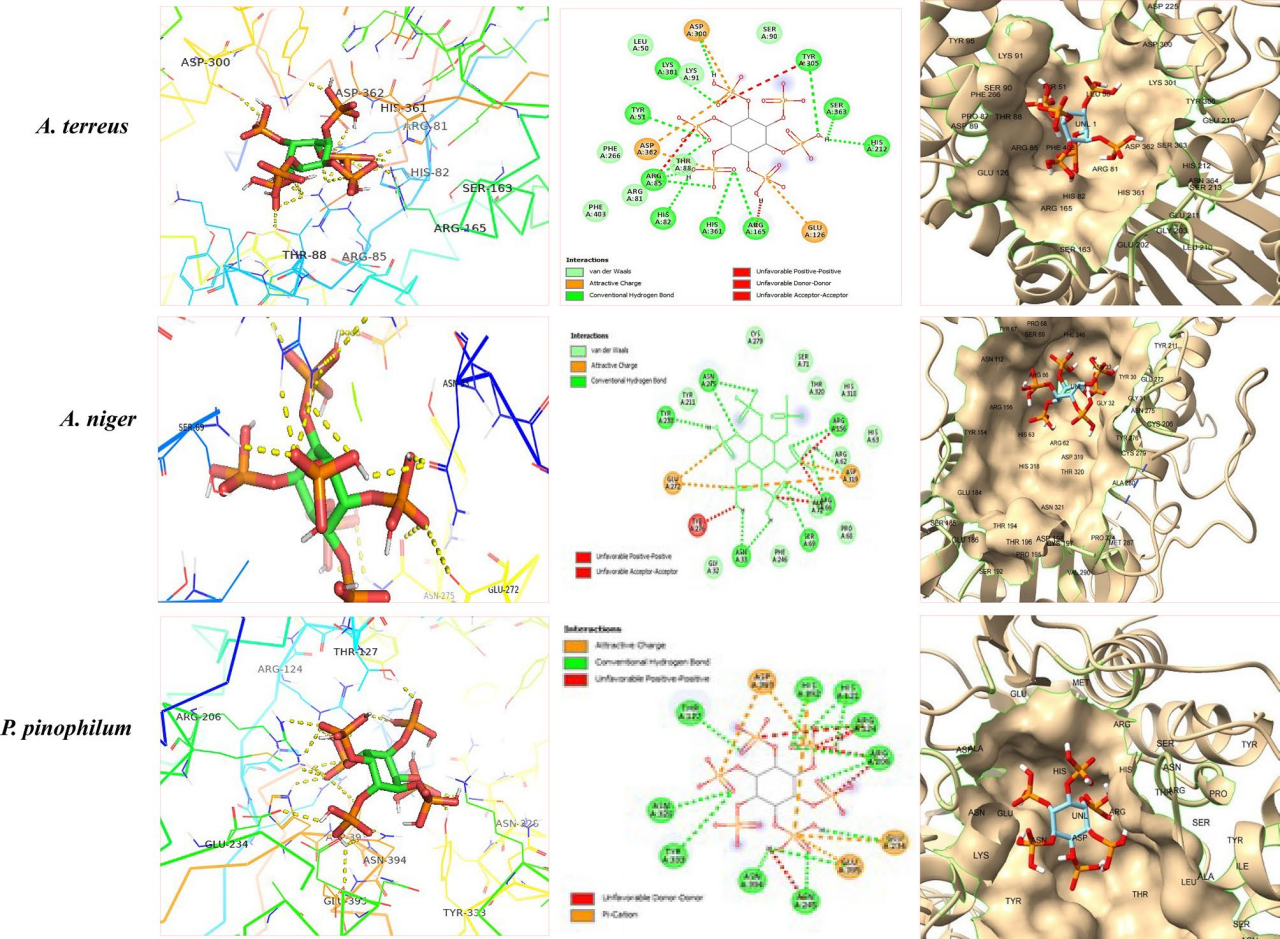
Molecular docking analysis of phytase from *Aspergillus terreus, A. niger* and *P. pinophilum* with phytic acid, via the hydrogen bond interactions (green dashed Line), van der Waals Interactions (yellow dotted line), and charge interactions (orange solid line)

### Molecular docking analysis of fungal phytases with phytic acid

From the docking results, diferent amino acids were involved in binding with phytic acid: hydrogen bond interactions with ARG362 and ARG81, van der Waals Interactions with LEU50, SER90, TYR305, PHE266, and PHE403, and attractive charge interactions with ASP301 and ASP362 (Fig. 6). The binding affinity of phytase of *A. terreus* with phytic acid was − 7.1 kcal/mol, ensures the stability of the interaction, while the binding energy of interactions of *A. niger* phytase with phytic acid was - 6.7 kcal/mol. The interactions of hytic acid with the enzyme includs both hydrogen and hydrophobic bonds, via the active site residues Arg62, His63, Arg66, Asp75, Arg156, Glu272 and His318. Residues Glu272 act as substrate specificity sites, while other residues act as catalytic sites. The binding interactions of *P. pinophilum* phytase with phytic Acid, reveals diverse molecular interactions, including appealing charges, traditional hydrogen bonds, and particular amino acid residues implicated in the binding process. Residues including threonine (THR A 127), aspartic acid (Asp A 393), and arginine (Arg A 124) are pivotal, whereas adverse interactions are observed, offering insights into the enzyme’s binding kinetics. So, from the molecular docking analyses, phytase from *A. terreus* had a significant efficiency and stability in hydrolyzing phytic acid, as revealed from the lower binding energy − 7.1 kcal/mol compared to *A. niger* and *P. pinophilum* (−6.7 to − 6.8 kcal/mol).

## In conclusion

Phytic acid is one of the main anti-nutritional compounds in animal feeds, for chelating of metal ions and amino acids, so, phytase is the powerful enzyme in hydrolyzing phytic acid by releasing the phosphorous in feed. However, the availability and stability of this enzyme are the major challenges, so, purification of thermostable phytase with unique biochemical properties was the objective. *Aspergillus terreus* EFBL-AS PV412881.1 was the potent phytase producer, with the highest enzyme productivity by growing on wheat bran, under solid state fermentation conditions optimized by the FCCD. The enzyme was purified to its molecular homogeneity by gel-filtration and ion-exchange chromatography, with 3.48 purification folds, with molecular subunit structure of 85 kDa. The maximum enzymatic activity was reported at 37–40 °C, and reaction pH 7.0, with slightly resistance to metal ions as inhibitors. The enzyme had a relative thermal stability by 5.2 h at 40 °C, with slightly lower thermal denaturation rate, than the previously purified phytases.

The phytic acid contents of the wheat bran were reduced by ~ 6.5 folds upon phytase treatment, ensuring the feasibility of this enzyme in the animal feed application, without affecting by the feed chemical components. From the molecular docking analysis, *A. terreus* phytase from had a higher affinity to hydrolyze phytic acid as revealed from the chemical interactions with lower binding energy − 7.1 kcal/mol, compared to that of *A. niger* and *P. pinophilum o*f − 6.7 kcal/mol, ensuring the stability of the interaction. Thus, for the availability and stability of *A. terreus* phytase, it could be used as influential ingredients in the animal feed to hydrolyze phytic acid *in situ*.

## Data Availability

All the data are provided in the manuscript.
